# A case series study of fever-diarrhea-hematochezia triad as the first presentation in plasma EBV DNA-positive diseases

**DOI:** 10.3389/fcimb.2026.1916422

**Published:** 2026-08-12

**Authors:** Huipeng Zhang, Xiaojun Song, Ying Liu, Huijun Chang, Xia Xu, Yanbo Yu, Yan Zhang

**Affiliations:** Department of Gastroenterology, Qilu Hospital of Shandong University, Jinan, Shandong, China

**Keywords:** differential diagnosis, EBER-ISH, Epstein-Barr virus, fever-diarrhea-hematochezia triad, lipid metabolism

## Abstract

**Aims:**

To characterize the clinical spectrum and outcomes of fever-diarrhea-hematochezia triad as the first presentation in plasma EBV DNA-positive diseases (EBV-FDH).

**Methods:**

This retrospective case series study was conducted at Qilu Hospital of Shandong University. We identified patients aged ≥14 years presenting with the triad of fever (>38 °C), diarrhea (≥3 loose stools/day), and hematochezia, accompanied by plasma EBV DNA positivity (≥500 copies/mL). All cases underwent a standardized diagnostic protocol including comprehensive laboratory tests, imaging, endoscopic and histopathological evaluation. Final diagnoses were established through multidisciplinary consensus.

**Results:**

The cohort (mean age 46.5 years; 71.4% male) frequently had immune abnormalities (50%). Lymphadenopathy (78.6%) and weight loss (50%) were common. Laboratory examinations revealed universal hypocholesterolemia, with reduced ApoB and elevated Lp(a)​ further characterizing the lipid-metabolic disturbance. Endoscopy revealed widespread ulcers. Diagnoses included CAEBV, ENKTL, IBD, etc. Misdiagnosis occurred in 57.1% of cases. Treatments varied (corticosteroids, chemotherapy, surgery). Treatment strategies were heterogeneous. Despite high overall mortality (64.3%), five patients achieved long-term survival, including one who received intensive therapies such as hematopoietic stem cell transplantation and combination chemotherapy, suggesting a potential benefit of aggressive intervention in selected cases.

**Conclusions:**

EBV-FDH is a severe syndrome with high mortality, often misdiagnosed initially. A high clinical suspicion and multimodal evaluation are crucial. Aggressive treatment, including surgery and HSCT, can enable survival in select patients.

## 1 Introduction

Epstein-Barr virus (EBV) is a widely distributed oncogenic virus and infects over 95% of world’s population (Abo et al., 1982; Cohen et al., 2011; Dunmire et al., 2018; Damania et al., 2022). Primary infection is usually asymptomatic or presents as an upper respiratory tract infection, but often leads to infectious mononucleosis (IM), characterized by sore throat, fever and cervical lymphadenopathy (Vedham et al., 2015; Dunmire et al., 2018). However, immunocompromised hosts (e.g., transplant recipients, HIV-infected individuals), those with underlying inborn errors of immunity (IEIs), or patients with specific EBV-associated pathologies (e.g., chronic active EBV infection [CAEBV], EBV-driven lymphomas) often develop severe, atypical disease presentations that diverge from this conventional clinical spectrum (Straus, 1988; Kawa, 2000; Heslop et al., 2010; Kerr, 2019; Prockop et al., 2020). Notably, IEIs are critical determinants of severe EBV pathogenesis; while some IEIs confer broad susceptibility to multiple pathogens, others result in a selective, high-penetrance (50-100%) vulnerability specifically to EBV (Latour and Winter, 2018; Sacco et al., 2023; Poli et al., 2025). Therefore, investigating IEIs in patients presenting with complicated EBV disease is essential for accurate diagnosis and targeted management. In these high-risk groups, EBV reactivation can lead to aggressive lymphoproliferative disorders (LPDs), systemic organ infiltration, or hemophagocytic lymphohistiocytosis (HLH) (Janka et al., 1998; Das et al., 2016). Notably, while gastrointestinal manifestations such as diarrhea, hematochezia, and colitis occur less frequently than classical IM symptoms, their presence often signifies serious underlying pathology. This pathology encompasses not only lymphomatous involvement, CAEBV-related organ damage, and LPDs progression (Shannon-Lowe et al., 2017; Allen and Preiksaitis, 2019; Kawada et al., 2023), but also IEIs. IEIs such as monogenic inflammatory bowel diseases and enteropathy associated with common variable immunodeficiency (CVID) can present with refractory diarrhea, severe gastrointestinal ulceration, and malabsorption (Van Schewick et al., 2020; Ahmed et al., 2025; Ghosh and Samanta, 2025; Juliana et al., 2025; Orange et al., 2026). When such IEI-related intestinal inflammation coincides with EBV infection or reactivation, it may closely mimic the clinical phenotype of EBV-FDH, posing a substantial diagnostic challenge. Therefore, a thorough evaluation for underlying immune defects through targeted genetic and immunological investigations is critical for accurate diagnosis and appropriate management. These gastrointestinal presentations warrant particular clinical attention as they may herald advanced disease and portend poorer outcomes.

Despite growing recognition of EBV’s diverse clinical manifestations, the specific triad of fever, diarrhea and hematochezia as the first presentation of plasma EBV DNA-positive disease remains poorly characterized in the medical literature. This symptom complex presents substantial diagnostic challenges, with potential etiologies spanning from EBV-driven colitis and direct viral mucosal invasion to lymphomatous infiltration or systemic complications of CAEBV/PTLD (Rahier et al., 2014; Shannon-Lowe et al., 2017; Allen and Preiksaitis, 2019). Critical knowledge gaps persist regarding the pathophysiological mechanisms underlying this clinical presentation, whether mediated by direct viral cytopathic effects, host immune responses, or neoplastic transformation. Furthermore, the approach to this distinct patient population is still an unmet clinical need.

Hereby, we summarized the demographic and clinical characteristics, treatment and prognosis of 14 cases of the fever-diarrhea-hematochezia triad as the first presentation in plasma EBV DNA-positive diseases (EBV-FDH), which seek to facilitate timely diagnosis, streamline diagnostic processes and refine management strategies for this high-risk population with potentially severe EBV-related manifestations.

## 2 Methods

### 2.1 Study design and setting

This retrospective case series study was conducted at Qilu Hospital of Shandong University. This study was granted an exemption from ethical review and the requirement for informed consent by the Ethics Committee on Scientific Research of Shandong University Qilu Hospital. Patient data were anonymized prior to analysis to ensure confidentiality.

### 2.2 Study population

All cases in this research were identified from the database of the Qilu Hospital of Shandong University from January 2016 to July 2025. We identified patients aged ≥14 years with the triad of fever (>38 °C), diarrhea (≥3 loose stools/day), and hematochezia (visible blood or strongly positive occult blood), accompanied by plasma EBV DNA positivity (≥500 copies/mL). Exclusion criteria included: established alternative diagnoses at initial presentation (e.g., inflammatory bowel disease, infectious colitis); incomplete diagnostic evaluation; or mild, self-limited symptoms without adequate investigation.

To facilitate comparative contextualization of the distinctive EBV-FDH phenotype, we additionally included 5 contemporaneous patients diagnosed with conventional EBV-associated intestinal lymphoma and 5 contemporaneous patients with EBV-negative inflammatory bowel disease (IBD). To further support quantitative differential diagnostic analyses in the Discussion, we also incorporated 5 contemporaneous cases each of IBD, intestinal tuberculosis (ITB) and Behçet’s disease (BD), all derived from the same institutional database and meeting established diagnostic criteria.

### 2.3 Data collection procedures

Clinical data were systematically extracted from electronic medical records using a standardized case report form. Trained researchers collected demographic information, clinical symptoms, laboratory examinations (including immunological profiles [serum immunoglobulin levels, lymphocyte subsets, systemic inflammatory markers, and EBV-specific testing], screening for co-infections, lipid profile, blood count, liver and renal function, coagulation profile), imaging and endoscopic records, pathological findings, surgical intervention, therapeutic regimen and outcomes. Genetic testing for inborn errors of immunity was not routinely measured in patients due to the retrospective design. All endoscopic images and pathology slides were re-reviewed by independent specialists to confirm findings.

The immune status of patients was categorized as “abnormal” based on the presence of predefined conditions known to cause immunosuppression prior to the onset of the EBV-FDH. These conditions included autoimmune diseases requiring immunosuppressive therapy, primary immunodeficiencies, a history of active or previously treated malignancy (excluding non-melanoma skin cancer), solid organ or hematopoietic stem cell transplant status, HIV infection, or the use of immunosuppressive medications (e.g., corticosteroids, biologics, chemotherapeutic agents) for any reason. The specific conditions for each patient categorized as having an “abnormal” immune status are available from the corresponding author upon reasonable request. A “normal” immune status was assigned to patients without any of these conditions prior to presentation.

### 2.4 Diagnostic evaluation

The diagnostic workup included comprehensive laboratory testing, contrast-enhanced abdominal imaging, and complete endoscopic evaluation with biopsy. Histopathological assessment incorporated routine staining, immunohistochemistry, and EBV-encoded RNA (EBER) *in situ* hybridization for EBV detection. Final diagnoses were established through multidisciplinary consensus involving gastroenterologists, infectious disease specialists, and pathologists.

### 2.5 Statistical analysis

Continuous variables were expressed as mean ± standard deviation or median (interquartile range) based on distribution normality. Categorical variables were presented as frequencies and percentages. Tests performed included the unpaired two-tailed Student’s t-test and Fisher’s exact test. Pearson’s correlation was performed to analyze the correlation. Survival probabilities were estimated using the Kaplan-Meier method, with differences between groups assessed by the log-rank test. To quantify the mortality risk associated with the EBV-FDH triad, univariate Cox proportional hazards modeling was performed, benchmarking against EBV-negative inflammatory bowel disease control cohort. A two-tailed *p* < 0.05 was defined as statistically significant. All analyses were performed using SPSS (version 29.0.1.0).

## 3 Results

### 3.1 Demographic and clinical features

Among the 14 patients identified in this study (**Table 1**), ten were males and only four were females. The average age was 46.50 ± 18.75 years. Seven cases (7/14) had an abnormal immune status at presentation. The median time from onset to hospital admission was 1.0 (1.0-3.5) months. The median peak temperature was 39.30 (39.00, 39.85) °C. Intermittent fever (IF) was the most common pattern (12/14), while continued fever (CF) and remittent fever (RF) were each observed in one case. The median frequency of diarrhea was 4.00 (3.00, 4.25) times daily. Stools were most commonly described as mucous (6/14) or loose (5/14), with fewer cases of mucopurulent and bloody (2/14) or watery (1/14) stools. Nine cases (9/14) presented with severe hematochezia, while five (5/14) cases exhibited mild hematochezia; eight cases (8/14) had undergone transfusion therapy. The color of the hematochezia was most frequently maroon (11/14), followed by melena (2/14) and bright red (1/14). Intestinal perforation occurred in four patients (4/14), and intestinal obstruction in two (2/14). The most prevalent extra-intestinal manifestations included lymphadenopathy (11/14), weight loss (7/14) and hepatosplenomegaly (4/14); other manifestations included oral ulcers (2/14), skin rash (2/14), night sweating (2/14) and nose sniffles (1/14). The median plasma EBV-DNA load upon presentation was 1.88E + 04 (4.38E + 03, 9.83E + 04) copies/mL, demonstrating considerable interindividual variability. Initial misdiagnosis was common (8/14), with inflammatory bowel disease (including Crohn’s disease [CD] and ulcerative colitis [UC]) and infectious etiologies being the most frequent preliminary impressions before the establishment of a final EBV-associated diagnosis.

**Table 1:** Demographic and clinical features of 14 EBV-FDH patients.

Case	Age/sex	Immune status	Duration​(Months)	T_max_/type of fever	Frequency (times/d)/ Characteristics of diarrhea	Degree/color (hematochezia)	Extra-triad and GI manifestation	Plasma EBV-DNA (copies/ml)	Misdiagnosis	Diagnosis
1	52/M	Abnormal	1	39.0/IF	10/loose	Severe/Melena	Hepatosplenomegaly, oral ulcers, weight loss	3.42E+04	Infective enteritis	CAEBV
2	64/M	Normal	8	40.0/CF	2/loose	Severe/Maroon	Hepatosplenomegaly, lymphadenopathy	4.41E+03	–	ENKTL
3	25/F	Normal	1	39.0/IF	3/loose	Severe/Melena	Hepatosplenomegaly, lymphadenopathy, weight loss	4.28E+03	–	DLBC
4	50/M	Normal	1	38.7/IF	5/loose	Mild/Maroon	Lymphadenopathy, weight loss	8.74E+04	–	CAEBV, R**/**O lymphoma
5	18/M	Normal	1	39.2/IF	4/mucous	Mild/Maroon	Lymphadenopathy, nose sniffles	2.36E+04	IM	ENKTL
6	51/M	Abnormal	1	39.2/IF	4/mucous	Severe/Maroon	Lymphadenopathy	1.82E+05	Ulcerative Colitis	EBV-associated enteritis
7	47/F	Normal	13	40/IF	4/loose	Severe/Maroon	Lymphadenopathy, oral ulcers, weight loss	2.35E+03	–	Crohn’s disease
8	70/M	Normal	1	39.0/IF	20/watery	Severe/Maroon	Lymphadenopathy, weight loss	6.68E+03	Ischemic colitis​	Lymphoma
9	29/M	Abnormal	12	39.8/IF	3/mucous	Mild/Maroon	Weight loss, skin rash	5.77E+06	Behçet’s Disease	Ulcerative Colitis
10	64/M	Normal	0.7	39.1/IF	3/mucoid bloody	Severe/Maroon	Lymphadenopathy, weight loss	2.38E+04	Lymphoma (suspected)​	CAEBV
11	14/M	Abnormal	0.5	39.8/IF	2/mucous	Mild/Maroon	Night sweating, skin rash	0.78E+03	Intestinal tuberculosis	Crohn’s disease
12	48/M	Abnormal	1	39.4/IF	4/mucoid bloody	Severe/Bright red	Lymphadenopathy, night sweating	1.39E+04	–	ENKTL
13	73/F	Abnormal	1	39.5/IF	4/mucous	Severe/Maroon	Lymphadenopathy	1.24E+04	–	ENKTL
14	46/F	Abnormal	2	41.0/RF	3/mucous	Mild/Maroon	Hepatosplenomegaly, lymphadenopathy	1.31E+05	PTLD	ENKTL

EBV-FDH, plasma EBV DNA-positive diseases; Duration, months from onset to admission; T_max_, peak temperature; IF, intermittent fever; CF, continued fever; RF, remittent fever. Definition of “severe”: resulting in a decrease in hemoglobin of >20g/L or requiring a blood transfusion. GI, gastrointestinal; IM, infectious mononucleosis; CAEBV, chronic active Epstein Barr virus infection; ENKTL, extranodal NK/T-cell lymphoma; DLBC, diffuse large B-cell lymphoma; R**/**O lymphoma, Lymphoma could not be ruled out; PTLD, post-transplant lymphoproliferative disorder.

### 3.2 Laboratory examinations

The laboratory findings of the 14 patients are summarized in **Table 2**. With the exception of one case (1/14) exhibiting an elevated white blood cell count, all other cases (13/14) presented normal results. The median lymphocyte count was 1.16 (0.55, 1.91) × 10^^9^/L, and seven patients (7/14) exhibited lymphopenia while the other seven (7/14) had normal counts. The average hemoglobin level was 90.57 ± 27.03 g/L, with 10 patients (10/14) exhibiting levels below the normal range. The average platelet count was 206.79 ± 84.01 × 10^^9^/L, with only one patient (1/14) exhibiting an abnormality.

**Table 2:** Laboratory examinations of 14 EBV-FDH patients.

Case	WBC (10^^9^/L)	Lymphocyte (10^^9^/L)	Hb (g/L)	PLT (10^^9^/L)	CRP (mg/L)	PCT (ng/mL)	Serum ferritin (ng/uL)	LFT	RFT	ALB (g/L)	TC (mmol/L)	TG (mmol/L)	LDL-C (mmol/L)	HDL-C (mmol/L)	Lp(a) (mg/L)	ApoB (g/L)	LDH (U/L)	Coag. Panel	EBVCA-IgM	EBVCA-IgG	EBNA-IgG	EBEA-IgG	Detection of other pathogens
1	4.56	0.39	70	196	47.8	0.884	515	N	N	24.1	2.41	0.89	1.22	0.68	385	0.58	212	N	–	+	+	+	–
2	6.54	3.01	100	136	3.14	0.07	438	N	N	36.9	3.87	1.24	2.15	1.12	210	0.92	196	N	–	+	+	+	–
3	2.51	0.53	48	137	43.4	0.156	361	N	N	26.6	1.92	0.63	0.87	0.52	412	0.51	481	N	–	+	+	+	–
4	9.15	2.69	115	297	17.3	0.083	687	N	N	28.6	2.78	1.05	1.49	0.79	198	0.67	251	N	–	+	+	+	–
5	5.86	1.91	138	306	3.12	0.043	779	N	N	49.3	4.12	1.56	2.38	1.21	145	0.95	165	N	–	+	+	+	–
6	2.71	0.53	69	208	10.5	0.075	373	N	N	33.4	2.15	0.72	1.03	0.61	398	0.55	229	N	+	+	–	+	HBV
7	7.29	1.61	97	262	48.7	0.123	141	N	N	33.7	2.93	1.18	1.64	0.84	267	0.72	178	N	–	+	+	–	–
8	10.1	0.69	67	26	59.34	1.21	202	N	N	29.6	1.76	0.54	0.79	0.47	456	0.48	244	A	–	+	+	+	–
9	3.98	0.63	118	172	17.99	0.06	912	N	N	39.8	2.54	0.98	1.31	0.73	289	0.68	204	N	–	+	+	+	HBV
10	6.68	0.55	75	244	18.76	0.053	323	N	N	46	2.31	0.81	1.12	0.65	312	0.59	245	N	–	+	+	+	–
11	8.27	1.63	127	359	38.47	0.13	581	A^*^	N	32.9	3.45	1.32	1.97	0.98	178	0.83	225	A	–	+	+	–	MTB
12	5.46	0.7	62	173	50.63	0.066	394	N	N	28.1	2.08	0.69	0.94	0.58	421	0.52	159	N	–	+	+	+	MTB
13	3.88	1.62	83	173	2.66	0.02	141	N	N	33.6	2.67	1.07	1.42	0.77	203	0.74	197	N	+	+	+	+	HBV
14	8.04	1.91	99	206	95.31	2.11	637	N	N	30.1	1.89	0.51	0.76	0.44	502	0.46	507	N	–	+	+	+	–

EBV-FDH, plasma EBV DNA-positive diseases; WBC, white blood cell; Hb, hemoglobin; PLT, platelet count; CRP, C-reactive protein; PCT, procalcitonin; LFT, liver function test; RFT, renal function tests; ALB, albumin; TC, total cholesterol; TG, triglyceride; LDL-C, low-density lipoprotein cholesterol; HDL-C, high-density lipoprotein cholesterol; Lp(a), Lipoprotein(a); ApoB, Apolipoprotein B; LDH, lactate dehydrogenase; Coag. Panel, coagulation panel; EBVCA-IgM, Epstein-Barr virus capsid antigen immunoglobulin M; EBVCA-IgG, Epstein-Barr virus capsid antigen immunoglobulin G; EBNA-IgG, Epstein-Barr virus nuclear antigen immunoglobulin G; EBEA-IgG, Epstein-Barr early antigen immunoglobulin G; N, normal; A, abnormal; +, positive; -, negative; MTB, *Mycobacterium tuberculosis*; HBV, hepatitis B virus. ^*^Liver function test: mildly abnormal.

**Table 2:** (continued). Laboratory examinations of 14 EBV-FDH patients.

Case	IgG (g/L)	IgA (g/L)	IgM (g/L)	CD19^+^B (%)	CD19^+^CD27^+^B(% of B cells)	CD4^+^T (/uL)	CD8^+^T (/uL)	CD4^+^T/CD8^+^T ratio	NK cell (/uL)
1	22.5	5.1	1.2	18.5	15	225	165	1.36	54
2	5.8	3.2	0.2	2.1	5	420	280	0.97	43
3	4.2	1.1	0.3	1.8	4	318	212	1.5	61
4	18.9	4.8	1.5	12.2	18	682	568	0.49	42
5	24.1	6.2	3.5	8.5	22	789	711	0.34	64
6	3.9	0.4	0.2	3.2	8	227	303	0.75	85
7	16.8	5.5	1.8	10.8	25	964	536	1.39	161
8	6.5	2.8	0.3	1.5	3	257	433	0.6	39
9	15.2	4.5	1.6	11.5	28	237	393	0.61	99
10	19.4	5.0	1.4	4.2	6	239	311	0.77	21
11	5.1	0.3	0.1	0.9	2	400	800	1.16	60
12	4.5	1.2	0.2	2.8	4	525	175	2.99	32
13	6.2	2.0	0.3	2.0	5	476	524	0.89	78
14	3.5	0.5	0.1	0.6	1	444	556	0.21	206

EBV-FDH, plasma EBV DNA-positive diseases; CD4^+^T, CD4^+^ T cell; CD8^+^T, CD8^+^ T cell; CD19^+^B, total B Cells; CD19^+^CD27^+^B, memory B Cells. Reference ranges; IgG 7.0–16.0 g/L; IgA 0.7–4.0 g/L; IgM 0.4–2.3 g/L; CD4^+^ T cells 410–1590/μL; CD8^+^ T cells 190–1140/μL; CD19^+^ B cells 5–25%; CD19^+^CD27^+^ memory B cells 15–50%.

Markers of systemic inflammation were frequently elevated. The median C-reactive protein (CRP) level was 38.47 (10.22, 49.67) mg/L, and 11 patients (11/14) had CRP levels above the normal limit. Procalcitonin (PCT) elevation was noted in 6 patients (6/14), with two cases showing significant increases. Seven patients (7/14) exhibited elevated serum ferritin (SF) levels, with a mean SF concentration of 463 ± 295 ng/μL. Hypoalbuminemia (Albumin < 40 g/L) was present in 12 patients (12/14), with the average albumin level of 33.76 ± 7.19 g/L. Lipid profiles (total cholesterol [TC], triglyceride [TG], low-density lipoprotein cholesterol [LDL-C], high-density lipoprotein cholesterol [HDL-C]) were available for all patients, with 11/14 (78.6%) exhibiting hypocholesterolemia (TC < 3.0 mmol/L) and 12/14 (85.7%) showing reduced HDL-C levels. The mean TC was 2.63 ± 0.74 mmol/L, mean TG was 0.94 ± 0.31 mmol/L, mean LDL-C was 1.36 ± 0.51 mmol/L, and mean HDL-C was 0.74 ± 0.23 mmol/L. TC levels are negatively correlated with CRP (r=-0.653, *p* = 0.015). Lower TC levels were numerically associated with elevated PCT (r=-0.463, *p* = 0.096), though statistical significance was limited by small sample size. Additionally, lipoprotein(a) [Lp(a)] was elevated (>300 mg/L) in 4/14 patients (mean 312.57 ± 116.06 mg/L), while apolipoprotein B (ApoB) was reduced (<0.8 g/L) in 11/14 patients (mean 0.66 ± 0.16 g/L). Low ApoB correlated strongly with hypoalbuminemia (r=0.574, p=0.032) and high CRP (r=-0.656, p=0.011), suggesting that the observed dyslipidemia is consistent with a (malnutrition-inflammation complex syndrome) MICS-like lipid disturbance pattern. Serum lactate dehydrogenase (LDH) was elevated in 5 patients (5/14), with the median LDH level of 218.5 (191.5, 246.5) U/L.

Coagulation panels were largely normal, except for abnormalities noted in two cases (2/14). Liver and renal function tests were predominantly within normal limits for most patients, with only one case (1/14) showing mild liver function abnormalities.

Serological profiling for EBV revealed that all patients were positive for EBV capsid antigen IgG (EBVCA-IgG). Two patients (2/14) were positive for EBV capsid antigen IgM (EBVCA-IgM), suggesting active primary infection; Two patients (2/14) were positive for EBV virus capsid antigen IgG (EBVCA-IgG) and EBV virus nuclear antigen IgG (EBNA-IgG) but negative for EBVCA-IgM and EBV early antigen IgG (EBEA-IgG), suggesting past infection with possible reactivation; Ten patients (10/14) were positive for EBVCA-IgG, EBNA-IgG and EBEA-IgG with negative EBVCA-IgM, confirming EBV reactivation or chronic active infection, which consistent with the aggressive clinical course observed. Notably, patients with chronic active EBV (CAEBV) and lymphoma (Cases 2, 3, 5, 8 12, 14) belonged to the third serological group, highlighting the utility of combined EBV antibody profiling in risk stratification.

Co-infections conditions were identified in five patients (5/14), including concurrent hepatitis B virus (HBV) infection in three patients and detection of *Mycobacterium tuberculosis* (MTB) in two patients.

Serum immunoglobulin assessment revealed a dichotomy in immunoglobulin profiles. Eight patients (Cases 2, 3, 6, 8, 11, 12, 13, 14) exhibited hypogammaglobulinemia, with Case 11 and 14 showing markedly reduced IgG, IgA, and IgM levels, suggestive of a potential underlying humoral immunodeficiency (e.g., CVID) or severe consumption due to chronic inflammation and lymphoma. Conversely, six patients (Cases 1, 4, 5, 7, 9, 10) demonstrated polyclonal hypergammaglobulinemia, likely reflecting chronic antigenic stimulation from active EBV replication and systemic inflammation. Only Case 5 presented with elevated IgM, while the majority of patients with lymphopenia or lymphoma (e.g., Cases 2, 3, 8, 12, 13) exhibited diminished IgM levels, indicating potential B-cell dysfunction.

Prior baseline immunoglobulin levels preceding the current infectious complication were unavailable for most patients due to the retrospective design, precluding definitive distinction between congenital immunodeficiencies and secondary consumption. Applying the international consensus criteria for CVID, defined as low IgG plus low IgA and/or low IgM, reduced memory B cells, and the absence of profound CD4^+^ T-cell lymphopenia (Bonilla et al., 2016), we retrospectively screened the cohort. Case 11​ (14-year-old, Crohn’s-like phenotype) fulfilled all criteria for a “Probable” CVID diagnosis, exhibiting pan-hypogammaglobulinemia (IgG 5.1, IgA 0.3, IgM 0.1 g/L), near-absent memory B cells (2%), and preserved CD4^+^ T-cell counts (400/μL). Additionally, Cases 6, 8, and 14​ exhibited profound hypogammaglobulinemia and B-cell depletion, warranting consideration of underlying humoral immune defects pending prospective genetic and immunological workup.

B-cell and memory B-cell phenotyping revealed that eleven (11/14) patients exhibited reduced B-cell counts, and this abnormality showed a certain degree of correlation with immunoglobulin levels: B-cell percentages were reduced in eight hypogammaglobulinemic patients, and memory B cells (CD19^+^CD27^+^) were nearly absent in patients with pan-hypogammaglobulinemia. Notably, Case 11 (14-year-old with Crohn’s-like presentation) exhibited pan-hypogammaglobulinemia, severe B-cell lymphopenia (0.9%), and near-absent memory B cells (2%), strongly suggesting an underlying IEI such as CVID.

T-cell subset analysis demonstrated a decreased CD4+/CD8+ T-cell ratio in seven patients (7/14). NK cell counts were generally below (11/14) the normal range. Absolute CD4^+^ and CD8^+^ T-cell counts were reduced in six of seven lymphopenic patients, with disproportionate CD8^+^ depletion in CAEBV/ENKTL cases and relative CD8^+^ predominance in inflammatory bowel disease-like phenotypes. NK cells were universally reduced in lymphopenic patients, with critical depletion in Cases 10 and 12.

### 3.3 Imaging findings

Positron emission tomography (PET) and computerized tomography (CT) were documented in ten patients, while four patients only received a CT scan without PET (**Table 3**). Segmental or diffuse intestinal wall thickening was commonly found in all cases. The spectrum of involvement ranged from localized segments to extensive tracts of bowel, frequently accompanied by mesenteric thickening, ascites, lymphadenopathy, and in one instance, a retroperitoneal mass. Large bowel involvement was observed in nine patients (9/14), small intestine involvement in ten (10/14), with five patients (5/14) exhibiting lesions in both large and small intestines. In patients undergoing PET, increased 18F-fluorodesoxyglucose (FDG) avidity was demonstrated in the affected intestinal segments and associated pathological structures. The pattern of extra-intestinal involvement was predominantly characterized by widespread lymph node enlargement across cervical, axillary, mediastinal, abdominal, and inguinal regions. Hepatic and splenic involvement was noted in seven patients (7/14), with additional instances of tonsillar, nasopharyngeal, bone marrow, renal, and adrenal gland uptake. Moreover, bone marrow involvement was noted in one patient (1/14). The maximal standardized uptake value (SUVmax) ranged from 3.9 to 21.5, with an average of 12.4, reflecting a generally high metabolic activity in these lymphoproliferative lesions.

**Table 3:** CT and/or PET features of 14 EBV-FDH patients.

Case	Affected bowel on CT	CT description	Extra-GI involvement on PET and/or CT	SUV max
1	Colon and ileum	Thickening of bowel wall	Abdominal lymph nodes, liver and spleen	7.8
2	Sigmoid colon and ileum	Mesenteric thickening, ascites and lymphadenopathy	Tonsils, nasopharynx, cervical, abdominal lymph nodes, liver and spleen	10.2
3	Colon and ileum	Thickening of bowel wall, retroperitoneal mass and lymphadenopathy	Abdominal lymph nodes, liver, spleen, kidney and adrenal gland	14.8
4	Colon and rectum	Thickening of bowel wall	Cervical, abdominal and inguinal lymph nodes	–
5	Ileum	Thickening of bowel wall and lymphadenopathy	Nasopharynx, cervical and abdominal lymph nodes, liver and spleen	12.3
6	Jejunum and ileum	Thickening of bowel wall, intestinal mass and lymphadenopathy	Abdominal lymph nodes	–
7	Jejunum and ileum	Thickening of bowel wall and lymphadenopathy	Abdominal and mesenteric lymph nodes	3.9
8	Colon and rectum	Thickening of bowel wall, ascites and lymphadenopathy	Abdominal lymph nodes, bone marrow, liver and spleen	20.7
9	Ileocecal region, sigmoid colon and rectum	Thickening of bowel wall	Mesenteric lymph nodes	–
10	Colon and rectum	Thickening of bowel wall	Cervical, axillary, abdominal and inguinal lymph nodes	9.5
11	Colon, rectum and ileum	Thickening of bowel wall	Ileocecal region and mesenteric lymph nodes	–
12	Jejunum	Thickening of bowel wall and lymphadenopathy	Nasopharynx and abdominal lymph nodes, liver and spleen	12.5
13	Jejunum and ileum	Thickening of bowel wall and lymphadenopathy	Tonsils, nasopharynx, abdominal and pelvic lymph nodes	10.6
14	Colon	Thickening of bowel wall, mesenteric thickening, ascites and lymphadenopathy	Nasopharynx, cervical, axillary, mediastinal, abdominal and pelvic lymph nodes, liver and spleen	21.5

PET, positron emission tomography; CT, computerized tomography; EBV-FDH, plasma EBV DNA-positive diseases; SUV max, average maximal standard uptake value.

### 3.4 Endoscopic examination

**Figure 1:**
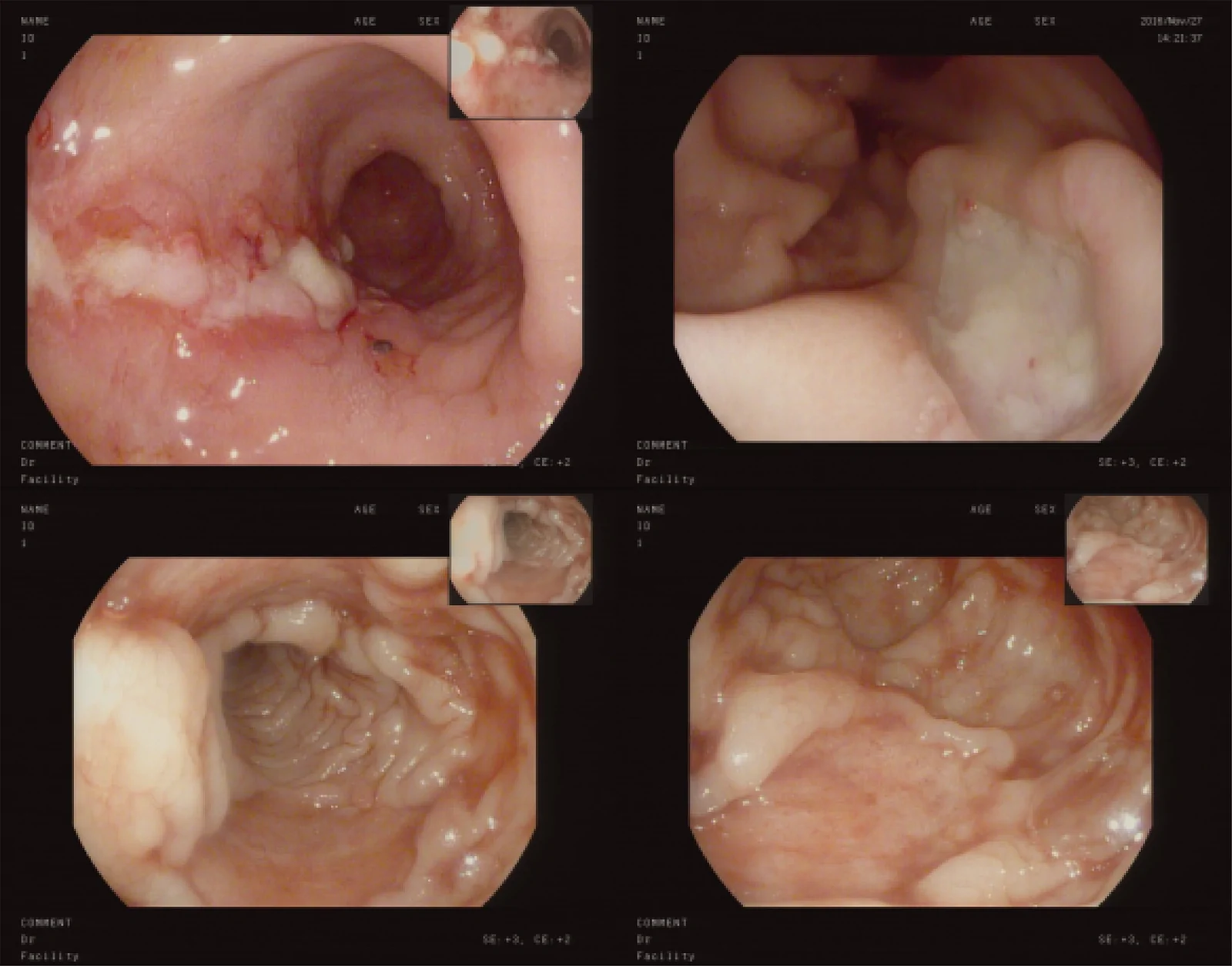
Endoscopic examination found large and deep ulcers in the intestinal wall with an oval or irregular shape.

All patients received digestive endoscopic examinations (**Table 4**). The endoscopic examination revealed multiple irregular ulcers of varying sizes and depths throughout the intestinal mucosa as the most predominant finding (**Figure 1**). These ulcers were frequently accompanied by mucosal edema, erosion and prominent nodules. In severe cases, large ulcers appeared turbid and hemorrhagic. The morphology of the ulcers was diverse, ranging from shallow, diminutive lesions resembling “mouse-bites” to large, deep ulcers extending to the muscular layer. Rigidity and stenosis of the intestinal wall were observed in a subset of patients. The involvement of the gastrointestinal tract was extensive and varied among the patients. The colon (11/14) was the most frequently affected segment, followed by the ileum (10/14) and rectum (5/14). A notable proportion of cases (7/14) exhibited diffuse lesions involving both the small intestine and colon. A cobblestone appearance was documented in three cases, two of which were diagnosed as CD. Active bleeding or oozing​ from the mucosal lesions was documented in three patients, indicating the severity of the vascular or mucosal injury. Histopathological examination of the endoscopic biopsy specimens revealed a spectrum of findings. The most common diagnosis was ​acute and/or chronic inflammation​ (9/14). ​Chronic inflammation with lymphoid hyperplasia​ was reported in three patients. Notably, ​NK/T-cell lymphoma (NKTL)​​ was confirmed in two patients. All but one patient (negative) showed a positive signal for Epstein-Barr virus-encoded small RNA *in situ* hybridization ​(​EBER-ISH).

**Table 4:** Endoscopic characteristics of 14 EBV-FDH patients.

Case	Involved locations	Profound ulcer	Shallow ulcer	Mucosal erosion	Mucosal edema	Intestine wall rigidity	Lumen stenosis	Cobblestone appearance	Active bleeding or oozing	Endoscopic pathology	EBER-SIH
1	Colon and ileum	Yes	Yes	Yes	Yes	No	No	No	No	Acute and chronic inflammation	Positive
2	Sigmoid colon and ileum	Yes	Yes	Yes	Yes	Yes	No	No	No	Acute and chronic inflammation	Positive
3	Colon, jejunum and ileum	Yes	Yes	Yes	Yes	Yes	No	No	Yes	NKTL	Positive
4	Colon and rectum	Yes	Yes	Yes	Yes	Yes	Yes	No	No	Severe acute and chronic inflammation	Positive
5	Colon and ileum	Yes	Yes	Yes	Yes	No	Yes	No	No	Chronic inflammation with lymphoid hyperplasia	Positive
6	Jejunum and ileum	No	No	No	Yes	No	No	No	No	Acute inflammation	Positive
7	Colon, jejunum and ileum	Yes	Yes	No	No	Yes	Yes	Yes	No	Severe chronic inflammation	Positive
8	Colon and rectum	Yes	Yes	Yes	Yes	Yes	Yes	No	No	Chronic inflammation with lymphoid hyperplasia	Positive
9	Esophagus, ileum, sigmoid colon and rectum	No	Yes	Yes	Yes	No	No	No	No	Acute and chronic inflammation	Positive
10	Colon and rectum	No	Yes	Yes	Yes	No	No	No	No	Acute and chronic inflammation	Positive
11	Colon, rectum and ileum	Yes	Yes	Yes	No	Yes	Yes	Yes	No	Acute and chronic inflammation	Negative
12	Stomach, jejunum and ileum	Yes	Yes	Yes	No	Yes	Yes	Yes	Yes	Acute and chronic inflammation	Positive
13	Ileum	No	No	Yes	Yes	No	No	No	No	NKTL	Positive
14	Stomach and colon	Yes	Yes	Yes	Yes	Yes	Yes	No	Yes	Chronic inflammation with lymphoid hyperplasia	Positive

EBV-FDH, plasma EBV DNA-positive diseases; EBER-SIH, *in situ* hybridization for Epstein-Barr virus-encoded small RNA; NKTL, NK/T-cell lymphoma.

### 3.5 Surgery and pathology diagnosis

Eight patients received surgical procedures in the disease course due to intestinal complications. Case 2, 3 and 13 experienced ileal perforation during the disease course; case 7 received right hemicolectomy with colostomy due to multiple colonic perforations; Case 8 and 14 received left hemicolectomy with colostomy due to intestinal obstruction; Case 9 received resection of the whole colon with ileostomy; case 12 underwent part of small intestine with ileostomy because of intractable gastrointestinal bleeding after chemotherapy. Surgery for case 8 was performed in a local hospital where the pathology report did not clearly diagnose the subtype of lymphoma. Case 4 did not undergo further diagnosis or treatment. Case 5 was diagnosed by cervical lymph node biopsy. Endoscopic pathology studies revealed “acute and chronic inflammation” in other three patients (cases 1, 10 and 11) and showed “acute inflammation” in case 6.

### 3.6 Treatment and outcome

The treatment and outcome of these 14 patients are summarized in **Table 5**. Their therapeutic approaches were diverse, including immunomodulatory agents (human immunoglobulin, 2/14), corticosteroids (10/14), antiviral therapy (ganciclovir, 10/14), and lymphoma-oriented chemotherapy regimens (7/14). The chemotherapeutic protocols employed were SMILE (steroid, methotrexate, ifosfamide, L-asparaginase and etoposide; 4/14), P-GemOx (pegaspargase, gemcitabine and oxaliplatin; 1/14), and R-CHOP (Rituximab, cyclophosphamide, doxorubicin, vincristine and prednisone; 1/14). Additionally, case 1 developed HLH later and received the ED (etoposide and dexamethasone) regimen, and case 12 received a PD-1 antibody. Surgical intervention was required in eight patients, primarily for severe complications such as intestinal perforation (4/8), intestinal obstruction (2/8), gastrointestinal bleeding (1/9), and toxic megacolon (1/9). The corresponding procedures included various forms of intestinal resection with ostomy. By the end of the follow-up, nine patients had died. The primary causes of death were multiple organ failure (4/9), sepsis (4/9), and HLH with hepatic failure (1/9). The median survival of deceased patients was 2 (1.5, 7.5) months. Five patients achieved long-term survival. Among them, three (Cases 6, 7 and 11) attained complete remission (CR) following standard treatment protocols. Notably, the longest survivor (Case 10) achieved prolonged survival after an intensive therapeutic regimen, which included induction with the SMILE chemotherapy protocol to partial remission (PR), followed by allogeneic hematopoietic stem cell transplantation (allo-HSCT).

**Table 5:** Diagnosis, treatment and outcome of 14 EBV-FDH patients.

Case	Tissue source of pathology diagnosis	Treatment	Response	Indication of surgery	Surgery type	Outcome	Cause of death	Survival (months)
1	Intestinal biopsy	Human immunoglobulin, corticosteroid and ganciclovir → ED	PR	–	–	Dead	HLH and hepatic failure	9
2	Resected intestine	Corticosteroid and ganciclovir → surgery →SMILE	PR	Ileal perforation	Ileectomy with ileostomy	Dead	Sepsis	12
3	Resected intestine	Corticosteroid → surgery → R-CHOP	PD	Ileal perforation	Ileectomy with ileostomy	Dead	Multiple organ failure	3
4	–	Human immunoglobulin, ganciclovir, mesalazine and symptomatic and supportive care	PD	–	–	Dead	Multiple organ failure	2
5	Lymph node	Symptomatic and supportive care	PD	–	–	Dead	Multiple organ failure	1
6	Intestinal biopsy	Corticosteroid, ganciclovir, Rituximab and mesalazine	CR	–	–	Survive	–	69
7	Resected intestine	Surgery → Corticosteroid, Infliximab and mesalazine	PR	Colonic perforations	Right hemicolectomy with colostomy	Survive	–	58
8	Resected intestine	Corticosteroid and ganciclovir → surgery → symptomatic and supportive care	PR	Intestinal obstruction	Left hemicolectomy with colostomy	Dead	Sepsis	2
9	Resected intestine	Corticosteroid, Vedolizumab and ganciclovir → surgery	PR	Toxic megacolon	Resection of the whole colon with ileostomy	Survive	–	34
10	Intestinal biopsy	SMILE and ganciclovir → allo-HSCT	CR	–	–	Survive	–	116
11	Intestinal biopsy	Corticosteroid, Ustekinumab and mesalazine	CR	–	–	Survive	–	33
12	Resected intestine	Surgery → SMILE and ganciclovir → PD-1	PD	Gastrointestinal bleeding	Part of small intestine with ileostomy	Dead	Sepsis	1
13	Resected intestine	Corticosteroid and ganciclovir → surgery → P-GemOx	PR	Ileal perforation	Ileectomy with ileostomy	Dead	Multiple organ failure	6
14	Resected intestine	Corticosteroid and ganciclovir → surgery → SMILE	PR	Intestinal obstruction	Left hemicolectomy with colostomy	Dead	Sepsis	2

EBV-FDH, plasma EBV DNA-positive diseases; ED, etoposide and dexamethasone; Allo-HSCT, allogeneic hematopoietic stem cell transplantation; SMILE, steroid, methotrexate, ifosfamide, L-asparaginase and etoposide; R-CHOP, Rituximab, cyclophosphamide, doxorubicin, vincristine and prednisone; HLH, hemophagocytic lymphohistiocytosis; PD-1, PD-1 antibody; P-GemOx, pegaspargase, gemcitabine and oxaliplatin; PR, partial remission; CR, complete remission; PD, progression of disease.

**Figure 2:**
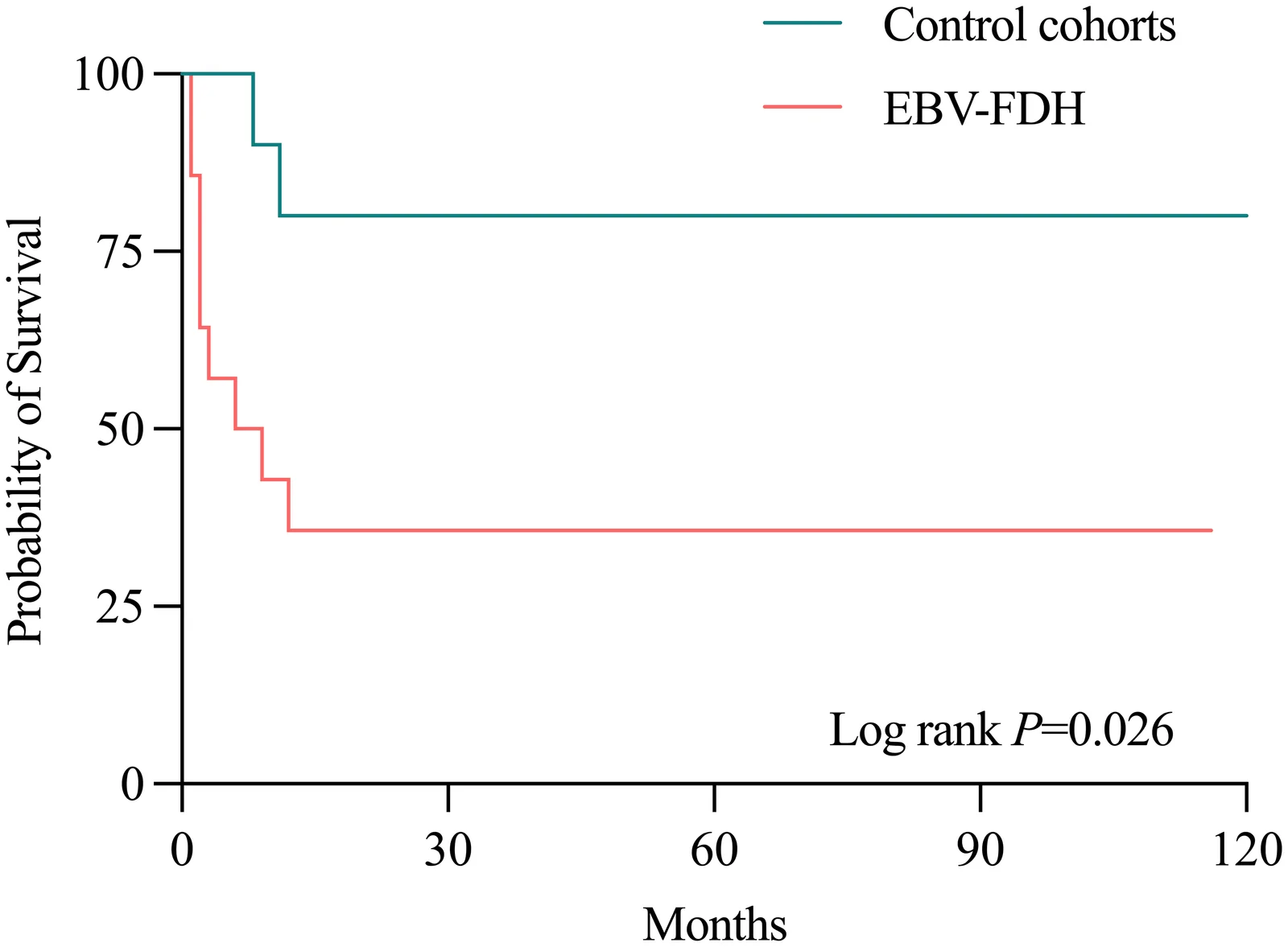
Kaplan-Meier analysis of overall survival comparing EBV-FDH with control cohort. A log-rank test was used to evaluate statistical differences.

Patients with EBV-FDH exhibited a median survival of 10.5 months, which was markedly shorter than that of control cohort (median survival of 72 months). To contextualize the dismal prognosis of EBV-FDH, we compared survival trajectories with contemporaneous control group (**Supplementary Table 1**). Kaplan-Meier analysis revealed a statistically significant divergence in survival curves between the EBV-FDH cohort and the combined control cohort (Log-rank *p* = 0.026)​ (**Figure 2**). Although the survival curve of conventional EBV-associated intestinal lymphoma controls showed a similar downward trend to EBV-FDH in the early phase, the overall survival probability of the control group stabilized at 80% after 11 months, whereas the EBV-FDH group continued to decline, reaching a plateau at approximately 36% survival. Univariate Cox proportional hazards modeling indicated that the presence of the fever-diarrhea-hematochezia triad conferred a significantly increased mortality risk (Hazard Ratio [HR]: 4.781, 95% CI: 1.025-22.307, *P* = 0.023) compared to EBV-negative IBD controls.

## 4 Discussion

In this retrospective case series, we reported 14 patients of EBV-FDH. All patients exhibited severe gastrointestinal involvement, and a subset developed life-threatening complications such as intestinal perforation, obstruction, or hemorrhage, necessitating multidisciplinary management. The strict inclusion and exclusion criteria employed in this study minimized selection bias and enhanced the reliability of our case series analysis.

EBV is a ubiquitous oncogenic virus that typically causes self-limited IM in immunocompetent individuals. However, in immunocompromised hosts or those with specific susceptibilities, EBV can lead to a spectrum of lymphoproliferative disorders, including CAEBV, ENKTL and HLH (Heslop et al., 2010; Kerr, 2019; Shen et al., 2023). While classical symptoms such as fever and lymphadenopathy are well-recognized, gastrointestinal manifestations, particularly the triad of fever, diarrhea and hematochezia, are less commonly emphasized in the literature, despite their association with aggressive disease and poor outcomes (Xu et al., 2020; Kawada et al., 2023).

The demographic and clinical profile of our EBV-FDH cohort exhibits notable distinctions from classic descriptions of systemic CAEBV and EBV-associated lymphomas, supporting its potential as a distinct clinical subgroup. The majority of our patients were male (71.4%), with a median age of 46.5 years. This profile stands in stark contrast to the pediatric predominance consistently reported in systemic CAEBV. A study reported that EBV-associated T/NK-LPDs predominantly affect children and adolescents, with a mean onset age of 11.3 years (Kimura et al., 2003). Similarly, A study involving 100 cases of CAEBV confirmed that systemic CAEBV primarily affects younger individuals, with a median age of 21 years (Yonese et al., 2020). Furthermore, our cohort’s age distribution also differs from that of CAEBV enteritis, which predominantly affects young to middle-aged patients (Liu et al., 2019). The high mortality rate (64.3%) observed in our series further underscores the aggressive nature of this presentation. This aligns with the poor prognosis associated with CAEBV involving the gastrointestinal tract (Shen et al., 2023), but the specific triad as the initial presentation appears to define a particularly severe phenotype. These comparative analyses suggest that the EBV-FDH triad may delineate a unique clinical entity or a distinct, aggressive late-onset form of EBV-driven disease, warranting specific clinical attention.

The clinical presentation of EBV-FDH often mimics IBD (Na et al., 2013), intestinal tuberculosis or other infectious colitis, leading to frequent misdiagnosis and delayed treatment. In our series, eight patients were initially misdiagnosed. Notably, among the five long-term survivors, only Case 10 was definitively diagnosed with an EBV-driven lymphoproliferative disease (CAEBV), while others had IBD or UC. This highlights the critical challenge of distinguishing patients with aggressive EBV-associated diseases (such as CAEBV or EBV-associated lymphomas), who often have an extremely poor prognosis, from those with IBD/UC or other conditions that may mimic its presentation but have different management pathways and outcomes. This diagnostic challenge underscores the importance of integrating systemic symptoms (e.g., fever, weight loss, lymphadenopathy), serological EBV profiling and quantitative EBV DNA testing into the initial evaluation of patients with unexplained gastrointestinal symptoms. Furthermore, a thorough assessment of the patient’s immune status, including age, history of transplantation, HIV infection, autoimmune diseases and immunosuppressive medication use, is crucial. This assessment aids not only in establishing the correct diagnosis but also in determining prognosis and guiding treatment decisions, such as deciding whether to reduce immunosuppression or intensify chemotherapy in cases of confirmed EBV-driven lymphoproliferative disorders.

Endoscopic findings in EBV-FDH were heterogeneous, ranging from shallow erosions to deep, penetrating ulcers, often with concomitant edema, rigidity, or stenosis. The presence of diffuse or multifocal lesions involving both the small and large bowel should raise suspicion for an EBV-driven process, particularly when extra-intestinal involvement is evident on imaging. PET-CT proved valuable in revealing systemic disease burden, with high SUVmax values supporting a malignant or lymphoproliferative etiology.

**Figure 3:**
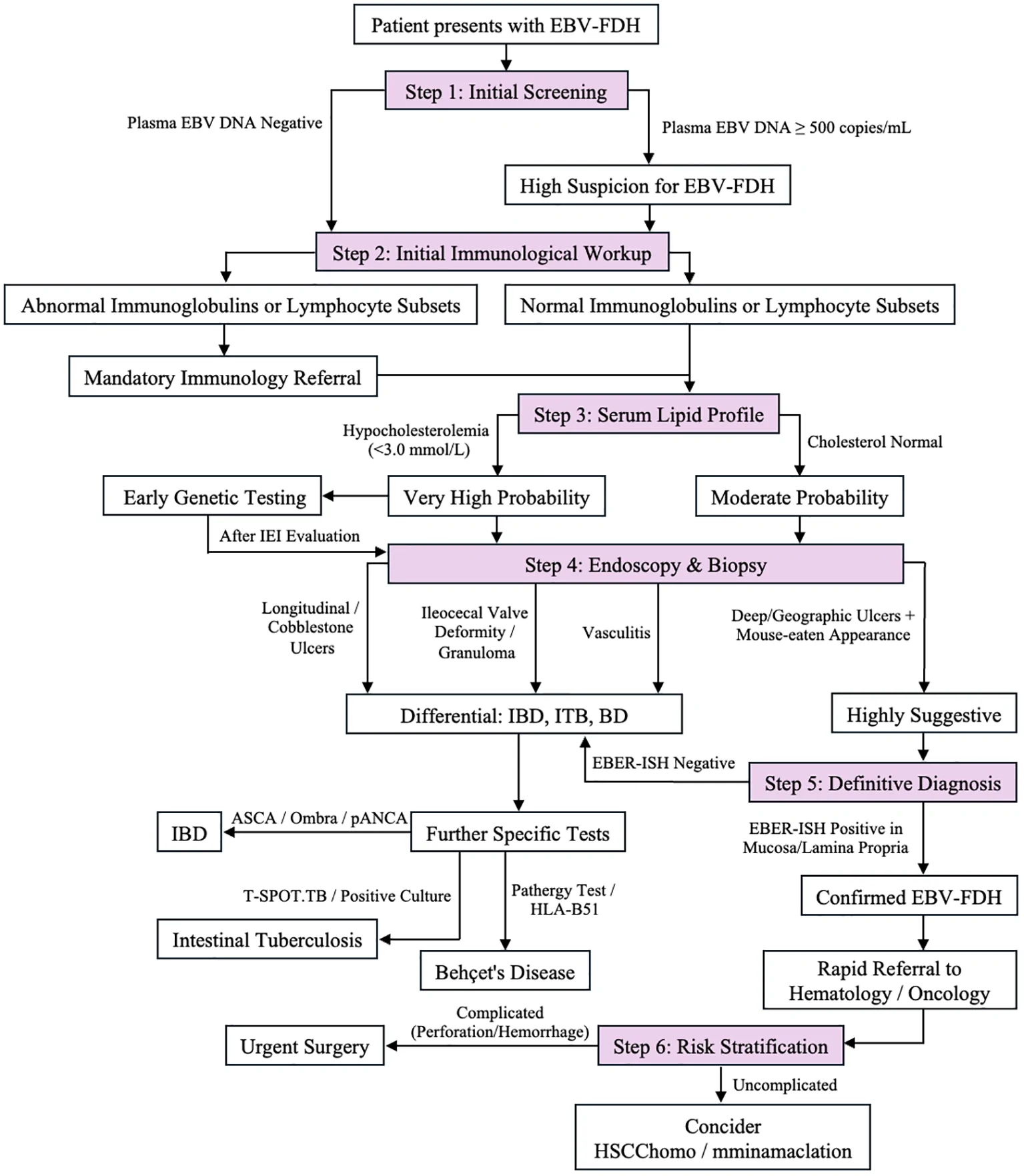
Proposed clinical workflow for patients presenting with the fever-diarrhea-hematochezia triad.​ The algorithm emphasizes rapid EBV DNA testing and lipid profiling as initial screening tools. Endoscopic appearance guides the differential diagnosis, but definitive diagnosis relies on EBER-ISH staining. Early recognition facilitates prompt referral for specialized oncologic or immunomodulatory therapy. ASCA, Anti-*Saccharomyces cerevisiae* antibodies; pANCA, Perinuclear anti-neutrophil cytoplasmic antibody.

Given the frequent misdiagnosis of EBV-FDH as other gastrointestinal disorders, we directly compared its key features with three common mimickers: IBD, ITB, and BD (**Table 6**). Unlike IBD, which typically lacks systemic EBV activation and shows chronic architectural distortion rather than deep penetrating ulcers, EBV FDH consistently presents with high plasma EBV DNA load (>500 copies/mL) and EBER ISH positivity in tissues. ITB often involves the ileocecal region with circumferential ulcers and caseating granulomas, whereas EBV FDH shows multifocal transmural ulcers with lymphoid hyperplasia or lymphomatous infiltration. BD features “punched out” ulcers and pathergy test positivity but rarely elevates EBV DNA or causes marked hypocholesterolemia. These distinctions underscore the value of integrating EBV serology, DNA quantification, and lipid profiling into the initial evaluation of patients with fever diarrhea hematochezia triad. Moreover, our comparative analysis reveals distinct quantitative discriminators in the four disease groups (**Supplementary Table 2**). Unlike controls, all EBV-FDH patients (100%) exhibited plasma EBV DNA ≥500 copies/mL and lacked granulomas or vasculitis on histology (all *P* < 0.001). While hypoalbuminemia was prevalent across groups, hypocholesterolemia (<3.0 mmol/L) was significantly more frequent in EBV-FDH (*P* = 0.002), serving as a rapid bedside screening tool. Furthermore, the median survival of EBV-FDH was drastically shorter than that of IBD, ITB or BD (*P* < 0.001), underscoring its aggressive nature. To operationalize these findings, we propose a simplified clinical algorithm (**Figure 3**)​ integrating EBV DNA screening, lipid profiling, and EBER-ISH confirmation to facilitate timely triage and risk-stratified management, thereby addressing the critical need for actionable diagnostic workflows in clinical practice.

**Table 6:** Key points for differentiating EBV-FDH from confusing diseases.

Feature	EBV-FDH	IBD	Intestinal tuberculosis	Behçet’s disease	IEIs (CVID/monogenic intestinal diseases with EBV susceptibility)
EBV DNA (plasma)​	≥500 copies/mL (all cases)	Negative/low-level	Negative	Negative	Often positive (≥500) in EBV-driven presentations
EBER-ISH​	Positive (13/14)	Negative	Negative	Negative	Often positive in affected tissues
Endoscopic ulcers​	Deep, irregular, multifocal; “mouse-bite” appearance	Superficial/linear (CD); continuous (UC)	Circumferential, transverse	“Punched-out,” oval	Variable: erosions to deep ulcers, often refractory
Lipid profile​	Marked hypocholesterolemia (78.6%)	Mild/none	Variable	Normal	Variable; may be normal or low
Systemic involvement​	Lymphadenopathy (78.6%), weight loss (50%)	Peripheral arthritis, uveitis	Pulmonary TB history	Oral/genital ulcers, uveitis	Recurrent infections, autoimmune cytopenias, lymphoproliferation
Response to steroids​	Poor unless EBV-targeted therapy added	Good	Poor (requires anti-TB)	Partial	Variable; often poor without Ig replacement/specific therapy
Key immunological clues	Plasma EBV DNA positivity	Normal immunoglobulins	Normal immunoglobulins	Normal immunoglobulins	Variable:​ May show pan-hypogammaglobulinemia (CVID) OR normal immunoglobulin levels​ (epithelial defects, CGD, IL-10 signaling defects, autoinflammatory disorders).
Genetic testing	Not performed	Not indicated	Not indicated	Not indicated	Recommended

EBV-FDH, plasma EBV DNA-positive diseases; IBD, inflammatory bowel disease; CD, Crohn’s disease; UC, ulcerative colitis; TB, *tuberculosis*; IEIs, nborn errors of immunity; CVID, common variable immunodeficiency.

Beyond IBD, intestinal tuberculosis, and Behçet’s disease, IEIs represent another critical group of mimickers that must be considered in patients presenting with the EBV-FDH triad (**Table 6**). IEIs such as CVID and monogenic disorders associated with EBV susceptibility can present with chronic diarrhea, severe gastrointestinal ulceration, lymphoproliferation, hepatosplenomegaly, lymphadenopathy, and a heightened risk of EBV-driven lymphomas and HLH (Nielsen and Lacasse, 2017; Van Schewick et al., 2020; Juliana et al., 2025). The overlap in clinical features between EBV-FDH and IEIs is substantial: both may manifest with fever, wasting, intestinal inflammation, and systemic lymphoproliferation. However, the presence of hypogammaglobulinemia, reduced B-cell or memory B-cell counts, and a family history of immunodeficiency or early-onset severe infections should prompt investigation for an underlying IEI (Sacco et al., 2023; Poli et al., 2025). Importantly, the severe, atypical EBV-related phenotype observed in our cohort, characterized by aggressive lymphoproliferative features, refractory intestinal ulcers and a high incidence of HLH, could in some cases signal an unrecognized IEI rather than a purely EBV-driven disease. Although we did not perform genetic testing for IEIs due to the retrospective nature of our study, we strongly recommend that future prospective evaluations of similar patients incorporate comprehensive immunophenotyping (including serum immunoglobulins, B-cell and T-cell subsets, and targeted gene panels) to rule out these potentially treatable underlying conditions. The frequent adult onset of CVID and its established association with an increased risk of lymphomagenesis (Bonilla et al., 2016) raise a critical question regarding our cohort: whether the aggressive EBV-driven lymphoproliferative disorders observed in some patients represent *de novo* malignancies or the terminal consequence of an undiagnosed, underlying IEI. Given that certain IEI patients exhibit impaired immune surveillance and chronic antigenic stimulation, they are exquisitely vulnerable to EBV-driven complications, including HLH and lymphoma. In our series, Cases 2, 8, 12, and 14 presented with both profound hypogammaglobulinemia and ENKTL/DLBCL. While pan-hypogammaglobulinemia is a known paraneoplastic phenomenon in lymphoma, the early age of onset in Case 11 and the severity of B-cell depletion across multiple cases suggest that IEI may have preceded and precipitated the lymphoproliferative process in select patients. Therefore, the EBV-FDH triad in a subset of adults may effectively unmask a previously silent primary immunodeficiency, highlighting the need for vigilance even in patients presenting in adulthood.

It is also noteworthy that immunoglobulin screening alone is insufficient for IEI detection in EBV-FDH-like presentations.​ Approximately 20% of all IEI genetic defects can manifest as bowel inflammation, and among the >80 monogenic causes of IBD-like intestinal disease, a substantial fraction, estimated at 30–40%, present with normal immunoglobulin levels​ (Pazmandi et al., 2019; Ouahéd, 2022; Hall and De Zoeten, 2024). This clinically important minority includes epithelial barrier defects, phagocyte defects (e.g., chronic granulomatous disease), IL-10 signaling defects, and autoinflammatory disorders (Gecse and Vermeire, 2018; Hall and De Zoeten, 2024). In our cohort, Cases 4 and 7 exhibited normal immunoglobulin levels yet experienced refractory disease courses, potentially representing such non-humoral IEIs. Current NASPGHAN, BSG/BSPGHAN, and the 2025 AAAAI/ACAAI/CIS IEI Practice Parameters all emphasize that normal serum immunoglobulin levels do not​ rule out underlying immune dysfunction (Kelsen et al., 2020; Kammermeier et al., 2023; Orange et al., 2026). Consequently, the EBV-FDH triad should trigger a broadened immunological and genetic workup, including neutrophil oxidative burst (DHR) testing, lymphocyte subset profiling, XIAP flow cytometry, and ultimately targeted genetic sequencing, rather than reliance on immunoglobulin measurement alone. Identifying an IEI would not only change the diagnostic label but also open avenues for specific therapies such as immunoglobulin replacement, targeted immunomodulation, or hematopoietic stem cell transplantation, which may improve outcomes.

Pathologically, the diagnosis of EBV-FDH relies on the demonstration of EBER by *in situ* hybridization in affected tissues. However, as seen in our and previous studies, endoscopic biopsies often yield nonspecific inflammatory changes (Shen et al., 2023), highlighting the limitations of superficial sampling and the need for deeper tissue acquisition or surgical resection in diagnostically challenging cases. Furthermore, the endoscopic appearance can be misleading, underscoring the critical importance of integrating pathological findings with the clinical picture. A notable example from our series is case 9 who presented with extensive ulcerations spanning from the esophagus to the rectum, an endoscopic pattern highly reminiscent of Behçet’s disease. However, the final diagnosis of ulcerative colitis was established through histopathological evaluation, which was not compatible with BD, in conjunction with the clinical course and response to therapy. This case exemplifies the significant overlap in endoscopic features among EBV-associated disorders, IBD, and other systemic conditions, and reinforces the necessity of a multimodal diagnostic approach that transcends endoscopic impression alone.

Beyond structural and inflammatory manifestations, our cohort revealed pronounced lipid disturbances, particularly hypocholesterolemia and reduced HDL-C, affecting over 70% of patients—a pattern increasingly recognized in severe viral infections and hematological malignancies (Castellanos-Castro et al., 2020; Liu et al., 2020; Guo et al., 2024). These alterations are unlikely to be incidental and likely reflect a convergence of host, viral, and disease-specific factors. First, systemic inflammation driven by EBV replication and cytokine storm (as evidenced by elevated CRP) upregulates IL-6 and TNF-α, which suppress hepatic cholesterol synthesis via HMG-CoA reductase inhibition and promote reverse cholesterol transport, thereby lowering circulating lipid levels (Zhang et al., 2015; Stefanutti et al., 2017; Górecka et al., 2022). Second, EBV may directly perturb host lipid homeostasis: the virus exploits lipid rafts for cellular entry and replicates within lipid-rich microdomains, increasing lipid consumption in infected cells (You et al., 2025). Third, chronic diarrhea, gastrointestinal ulceration, and malabsorption may further exacerbate lipid depletion through reduced nutritional intake and impaired absorption. Importantly, hypocholesterolemia has been independently associated with adverse outcomes in infectious and lymphoproliferative disorders, correlating with higher viral loads, greater disease burden, and increased mortality risk (Mogensen et al., 2020; Ozturk, 2021; Hofmaenner et al., 2023). Although our small sample size precluded formal prognostic modeling, the numerical trend linking lower total cholesterol to higher EBV DNA and CRP levels suggests that lipid parameters may serve as accessible, bedside-compatible biomarkers for risk stratification in EBV-FDH. Larger prospective studies are warranted to determine whether serial lipid monitoring can guide treatment intensity and predict therapeutic response. Moreover, the addition of Lp(a) and ApoB measurements refined our understanding of lipid perturbations in EBV-FDH. Elevated Lp(a), an independent cardiovascular risk factor, may reflect acute-phase reactant behavior during EBV-driven inflammation, while low ApoB aligns with impaired hepatic synthesis due to cytokine-mediated suppression (e.g., IL-6 inhibiting HMG-CoA reductase). These markers, combined with TC/HDL-C, could enhance risk prediction of higher mortality risk, warranting validation in larger cohorts.

The aggressive phenotype of EBV-FDH likely arises from the convergence of three interrelated pathological axes. First, EBV-induced direct mucosal injury​ initiates the clinical cascade. EBV not only infects B cells but also demonstrates tropism for intestinal epithelial cells and lymphoid aggregates within the lamina propria (Kimura et al., 2012; You et al., 2025). This viral cytopathy, coupled with EBER-driven oncogenic signaling, disrupts epithelial barrier integrity, leading to deep ulcerations, hemorrhage, and the characteristic “mouse-bite” endoscopic appearances observed in our cohort. Second, inflammation-driven lipid metabolic dysregulation​ explains the near-universal hypocholesterolemia and reduced ApoB levels (Stefanutti et al., 2017; Górecka et al., 2022). The chronic EBV antigen stimulation fosters a cytokine storm, evidenced by elevated CRP and PCT in our series, which suppresses hepatic HMG-CoA reductase activity via IL-6 and TNF-α signaling, thereby inhibiting cholesterol synthesis. Concurrently, systemic inflammation accelerates reverse cholesterol transport and promotes lipid catabolism, while severe diarrhea and malabsorption exacerbate nutrient depletion. The strong negative correlation between TC and CRP (r=-0.653, p=0.015) in our data substantiates this inflammation-lipid axis. Third, immune dysregulation facilitates lymphomagenesis, particularly in the context of underlying IEIs (Poli et al., 2025; Orange et al., 2026). As exemplified by Case 11, impaired immune surveillance due to NK-cell and CD8^+^ T-cell cytopenia, combined with persistent antigenic stimulation, creates a microenvironment conducive to clonal expansion of infected T/NK cells. This progression from reactive lymphoid hyperplasia to overt lymphoma (e.g., ENKTL in Cases 2, 5, 12, 13, 14) underscores the precarious balance between ineffective immune control and malignant transformation in EBV-FDH.

Given the marked heterogeneity in clinical presentations and outcomes, management of EBV-FDH necessitates a standardized, risk-stratified framework rather than fragmented empiricism: emergent surgical resection is prioritized for life-threatening complications (e.g., perforation, obstruction, refractory hemorrhage) to mitigate fatal acute events; pathological subtyping directs subsequent therapy, with upfront lymphoma-directed chemotherapy (e.g., SMILE for ENKTL) mandated for clonal lymphoproliferative disorders and stepwise immunosuppression reduction plus antiviral therapy reserved for CAEBV without overt malignancy; allogeneic HSCT represents the only curative option for eligible patients with chemosensitive disease, as evidenced by our longest survivor achieving 116-month remission, while PD-1 inhibitors show no clear survival benefit in this population based on our cohort data; finally, intravenous immunoglobulin replacement is recommended for patients with profound hypogammaglobulinemia or suspected inborn errors of immunity to reduce infection-related mortality during definitive anti-EBV therapy.

Consistent with this stratified framework, treatment responses in our cohort varied markedly by risk strata, including immunomodulators, antivirals, corticosteroids and lymphoma-directed chemotherapy. The five surviving patients received relatively standardized treatment protocols, whereas the therapeutic regimens for the nine deceased patients were highly heterogeneous. This variability may be attributed to the limited evidence in the existing literature and the patients’ critically poor prognosis, which often necessitated individualized, albeit suboptimal, approaches. Surgical intervention was necessary in over half of the patients, primarily for complications such as perforation or obstruction. Future management of such complex cases should emphasize enhanced multidisciplinary collaboration, particularly involving hematology-oncology specialists, to establish more uniform and evidence-based therapeutic strategies. Notably, the treatment courses of long-term survivors provide critical clues for future strategy development. Case 10, who achieved the longest survival (116 months), was successfully treated with the SMILE chemotherapy regimen followed by allo-HSCT. Meanwhile, Case 7 attained sustained remission after surgical intervention for colonic perforations, followed by post-operative immunosuppressive therapy. These experiences suggest that early, intensive, and multimodal treatment strategies may be pivotal for improving outcomes in selected patients.

Despite these efforts, the overall prognosis of EBV-FDH remains poor, with nearly two-thirds of patients succumbing to disease progression, sepsis, or multiorgan failure. This underscores the need for early diagnosis, risk stratification and novel therapeutic approaches. The successful use of PD-1 inhibitors and allo-HSCT in other EBV-associated LPDs offers promising avenues for future investigation (Kimura et al., 2012; Liu et al., 2020). Although the use of a PD-1 antibody in Case 12 did not prevent disease progression in our cohort, its investigation remains warranted, potentially in combination regimens or selected settings. Coupled with the success of allo-HSCT in Case 10, these modalities represent promising avenues worthy of further exploration.

This study has several limitations that should be acknowledged. First, its single-center, retrospective design inherently introduces potential selection and information biases. Second, the relatively small sample size, while reflective of the rarity of this specific presentation, limits the statistical power to identify robust prognostic factors, precluding both meaningful subgroup analyses and the execution of multivariable regression models to confirm the triad as an independent predictor of survival. Third, systematic genetic testing for IEI was not performed, which precluded definitive identification of underlying primary immunodeficiencies in patients presenting with severe phenotypes or HLH.​ Fourth, the presence of plasma cells in the lamina propria was not consistently documented across all biopsy specimens, and gastroscopy was not uniformly performed in the cohort, leaving the true prevalence of gastric involvement in EBV-FDH undefined and limiting comprehensive characterization of the disease’s gastrointestinal manifestations. Notably, this gap is particularly relevant for identifying underlying CVID: paucity of gastrointestinal plasma cells is a pathognomonic feature of this immunodeficiency, and standardized endoscopic evaluation (including both upper and lower endoscopy) is recommended for patients with suspected IEI to enable early detection of CVID-related pathology and mitigate long-term malignancy risk (Hashash et al., 2022; Marsden et al., 2024).​ Consequently, the generalizability of our findings and conclusions may be constrained. Future multi-center, prospective studies with larger cohorts are warranted to validate our observations and to definitively elucidate the clinical characteristics and optimal management strategies for this challenging patient population.

## 5 Conclusions

The triad of fever, diarrhea, and hematochezia as the initial presentation of plasma EBV DNA-positive disease represents a rare but clinically significant syndrome characterized by aggressive behavior and high mortality. Its nonspecific presentation often leads to misdiagnosis as IBD or infection. A high index of suspicion, combined with comprehensive serological, imaging, and histopathological evaluation, including EBER-ISH and mandatory systematic screening for underlying IEIs, is essential for timely diagnosis and treatment. Multimodal therapy, including surgery for complications, appears crucial for managing severe cases. While HSCT may offer a potentially curative option in selected patients, the role of PD-1 inhibitors remains uncertain, and further investigation is warranted before recommending this approach. Continued research into novel targeted and immunomodulatory strategies is urgently needed to improve outcomes in this challenging patient population.

## Data Availability

The raw data supporting the conclusions of this article will be made available by the authors, without undue reservation.
