# Supplementary material for: A case series study of fever-diarrhea-hematochezia triad as the first presentation in plasma EBV DNA-positive diseases
Source: Front Cell Infect Microbiol. 2026 Aug 12;16:1916422. doi: 10.3389/fcimb.2026.1916422 (PMC13506766; doi:10.3389/fcimb.2026.1916422)
Supplement: Supplementary file 1 [file Table1.docx]

**Supplementary Table 1. Clinical features and outcomes of control groups**

| Case | Diagnosis | Tissue source of  pathology diagnosis | Treatment | Response | Indication of surgery | Surgery type | Outcome | Cause of death | Survival (months) |
| --- | --- | --- | --- | --- | --- | --- | --- | --- | --- |
| EBV-associated Intestinal Lymphoma | | | | | | | | | |
| C1 | ENKTL | Resected intestine | SMILE → allo-HSC | CR | Intestinal perforation | Partial enterectomy + anastomosis | Survive | - | 48 |
| C2 | DLBCL | Intestinal biopsy | R-CHOP → radiotherapy | PR | Intestinal obstruction | Adhesiolysis + diverting stoma | Survive | - | 14 |
| C3 | CAEBV with LPD | Lymph node | Chemotherapy + prednisone | PD | Refractory hemorrhage | - | Dead | HLH | 8 |
| C4 | ENKTL | Resected intestine | Surgery → P-GemOx | PR | Perforation | Right hemicolectomy | Dead | Multiple organ failure | 11 |
| C5 | DLBCL | Intestinal biopsy | R-CHOP | CR | - | - | Survive | - | 60 |
| EBV-negative Inflammatory Bowel Disease | | | | | | | | | |
| C6 | CD | Intestinal biopsy | Corticosteroid + Azathioprine | CR | Stricturing disease | Strictureplasty | Survive | - | 117 |
| C7 | UC | Intestinal biopsy | 5-ASA + Infliximab | PR | Toxic megacolon | Subtotal colectomy + ileostomy | Survive | - | 120 |
| C8 | CD | Intestinal biopsy | Ustekinumab + Metronidazole | CR | Internal fistula | Fistula resection + primary repair | Survive | - | 116 |
| C9 | UC | Intestinal biopsy | Corticosteroid → Cyclosporine | PR | - | - | Survive | - | 90 |
| C10 | CD | Intestinal biopsy | Mesalazine + Budesonide | CR | - | - | Survive | - | 84 |

Abbreviations: ENKTL, extranodal NK/T-cell lymphoma; DLBCL, diffuse large B-cell lymphoma; CAEBV, chronic active Epstein-Barr virus; LPD, lymphoproliferative disorder; CD, Crohn's disease; UC, Ulcerative colitis; allo-HSCT, allogeneic haematopoietic stem cell transplantation; SMILE, steroid, methotrexate, ifosfamide, L-asparaginase and etoposide; R-CHOP, Rituximab, cyclophosphamide, doxorubicin, vincristine and prednisone; P-GemOx, pegaspargase, gemcitabine and oxaliplatin; 5-ASA, 5-aminosalicylic acid; CR, complete remission; PR, partial remission; PD, progression of disease; HLH, hemophagocytic lymphohistiocytosis.

**Supplementary Table 2.** Comparative clinical, laboratory, and endoscopic features among EBV-FDH, IBD, Intestinal Tuberculosis (ITB), and Behçet’s Disease (BD).

|  | EBV-FDH (n=14) | IBD (n=5) | ITB (n=5) | BD (n=5) | P value  (EBV-FDH vs. Others) |
| --- | --- | --- | --- | --- | --- |
| **Demographics** | | | | | |
| Age (years) | 46.50 ± 18.75 | 43.00 ± 13.27 | 44.00 ± 14.54 | 44.40 ± 5.77 | 0.637 |
| Male Sex, n (%) | 10 (71.4) | 3 (60.0) | 3 (60.0) | 4 (80.0) | 0.782 |
| **Key Laboratory Markers** | | | | | |
| Plasma EBV DNA ≥ 500 copies/mL, n (%) | **14 (100)** | 0 (0) | 0 (0) | 0 (0) | **<0.001** |
| Hypocholesterolemia (<3.0 mmol/L), n (%) | **11 (78.6)** | 1 (20.0) | 2 (40.0) | 0 (0) | **0.002** |
| CRP (mg/L) | 38.47 (10.22, 49.67) | 28.12 (13.84, 38.73) | 55.15 (42.10, 75.84) | 12.76 (7.20,25.51) | 0.928 |
| Hypoalbuminemia (Albumin < 40 g/L), n (%) | 12 (85.7) | 1 (20.0) | 3 (60.0) | 0 (0) | **0.001** |
| **Endoscopic Features** | | | | | |
| Profound Ulcers, n (%) | **10 (85.7)** | 2 (40.0) | 4 (80.0) | 3 (60.0) | 0.812 |
| **Histopathology** | | | | | |
| EBER-ISH Positive, n (%) | **13 (92.9)** | 0 (0) | 0 (0) | 0 (0) | **<0.001** |
| Granuloma, n (%) | 0 (0) | 0 (0) | **5 (100)** | 0 (0) | **<0.001** |
| Vasculitis, n (%) | 0 (0) | 0 (0) | 0 (0) | **4 (80.0)** | **<0.001** |
| **Clinical Outcome** | | | | | |
| Median Survival (months) | **9.0** | >84 | >105 | >120 | **<0.001** |

Abbreviations: ESR, erythrocyte sedimentation rate; CRP, C-reactive protein; EBER-ISH, Epstein-Barr virus-encoded small RNA in situ hybridization. *P*-values were calculated using unpaired two-tailed Student's t-test and Kruskal-Wallis test for continuous variables and Fisher's exact test for categorical variables, comparing EBV-FDH against the pooled control groups (IBD, ITB, BD).
